# Antifungal Susceptibility Patterns, *In Vitro* Production of Virulence Factors, and Evaluation of Diagnostic Modalities for the Speciation of Pathogenic *Candida* from Blood Stream Infections and Vulvovaginal Candidiasis

**DOI:** 10.1155/2014/142864

**Published:** 2014-07-07

**Authors:** Chaitanya Tellapragada, Vandana Kalwaje Eshwara, Ruqaiyah Johar, Tushar Shaw, Nidhi Malik, Parvati V. Bhat, Asha Kamath, Chiranjay Mukhopadhyay

**Affiliations:** ^1^Department of Microbiology, Kasturba Medical College, Manipal University, Manipal, Karnataka 576104, India; ^2^Department of Obstetrics and Gynecology, Melaka Manipal Medical College, Manipal University, Manipal, Karnataka 576104, India; ^3^Department of Community Medicine, Kasturba Medical College, Manipal University, Manipal, Karnataka 576104, India

## Abstract

*Candida* spp. have emerged as successful pathogens in both invasive and mucosal infections. Varied virulence factors and growing resistance to antifungal agents have contributed to their pathogenicity. We studied diagnostic accuracy of HiCrome *Candida* Differential Agar and Vitek 2 Compact system for identification of* Candida *spp. in comparison with species-specific PCR on 110 clinical isolates of* Candida *from blood stream infections (54, 49%) and vulvovaginal candidiasis (56, 51%).* C. albicans* (61%) was the leading pathogen in VVC, while* C. tropicalis *(46%) was prominent among BSIs. HiCrome Agar and Vitek 2 Compact had good measures of agreement (*κ*) 0.826 and 0.895, respectively, in comparison with PCR. We also tested these isolates for* in vitro* production of proteinase, esterase, phospholipases, and biofilms. Proteinase production was more among invasive isolates (*P* = 0.017), while phospholipase production was more among noninvasive isolates (*P* = 0.001). There was an overall increase in the production of virulence factors among non-*albicans Candida*. Identification of clinical isolates of* Candida* up to species level either by chromogenic agar or by Vitek 2 Compact system should be routinely done to choose appropriate therapy.

## 1. Introduction

The opportunistic yeasts belonging to the genus* Candida* have been associated with a wide range of human infections and significant mortality and morbidity. The clinical manifestations range from infections of the superficial skin and its appendages to deep-seated or disseminated candidiasis. Estimates suggest that* Candida* spp. have been the third most common nosocomial pathogens associated with blood stream infections (BSIs) [1]. Among noninvasive infections, vulvovaginal candidiasis (VVC) affects approximately 75% of the women with at least one episode during their lifetime [2]. Though* C. albicans* has been associated mostly with human infections, there has been an increase in the prevalence of infections due to non-*albicans Candida* in the recent past [3, 4]. Of the many pathogenic non-*albicans* species known,* C. tropicalis, C. parapsilosis, C. kefyr, C. krusei*, and* C. guilliermondii* are mostly associated with human infections.* Candida* spp. have grown from successful commensals to pathogens in various body sites with the help of many virulence determinants. Though non-*albicans Candida* have proven to be common pathogens in most invasive infections, little attention is given to their virulence attributes. Adherence to host tissue, response to environmental changes, secretion of hydrolases, and biofilm production are a few of the most important virulence mechanisms of these fungi [5]. The exact mechanism of candidal pathogenesis is of prime interest for many researchers globally. With the increasing rate of infections due to non-*albicans Candida* and varying susceptibilities to commonly used empirical antifungal agents like fluconazole, early and accurate species identification would help the clinician in befitting therapeutic management [6]. However, from a microbiologist's perspective, conventional methods like sugar assimilation, fermentation reactions, and identification based on morphological characters are tedious and time-consuming. This emphasizes the need for testing various work flow algorithms using commercially available diagnostic modalities to generate a rapid and accurate identification of* Candida* species.

The aim of this study was to evaluate the diagnostic utilities of commercial chromogenic medium and Vitek 2 Compact system against polymerase chain reaction (PCR) in species identification of clinical isolates of* Candida*. It was also aimed at detection and comparison of* in vitro* production of virulence factors among invasive and noninvasive (mucosal) clinical isolates of* Candida* and at studying their antifungal susceptibility patterns.

## 2. Materials and Methods

The present study was carried out on 110 clinical isolates of* Candida* spp. obtained from patients diagnosed either with blood stream infection or with vulvovaginal candidiasis during a period of one year (March 2012–February 2013) in the laboratory attached to a tertiary care teaching hospital in Southern India.

Two commercially available methods for the species identification of* Candida*, namely, HiCrome* Candida* Differential Agar (HiMedia, Mumbai, India) and Vitek 2 Compact system (bioMerieux, Marcy l' Etoile, France), were compared with species-specific PCR as the gold standard.

### 2.1. Comparison between HiCrome* Candida* Differential Agar, Vitek 2 Compact System, and* Candida* Species-Specific PCR

#### 2.1.1. HiCrome* Candida* Differential Agar

*Candida* isolates were streaked on HiCrome Agar plates as per manufacturer's instructions and incubated at 37°C for 48 hrs. The plates were examined at 24 hrs and 48 hrs for the colony morphotypes. At the end of 48 hrs, the final reading of all the plates was taken by two independent observers to determine the interobserver measure of agreement (*κ*).

#### 2.1.2. Vitek 2 Compact System

Fresh subcultures of all the* Candida* isolates were obtained on Sabouraud's dextrose agar (SDA) plates for identification and antifungal susceptibility testing using the Vitek 2 Compact, ID YST, and AFT (YS06) cards as per manufacturer's instructions. In YS06 cards, antifungal susceptibility results based on interpretative guidelines recommended by Clinical Laboratory Standards Institute (CLSI) 2012 were obtained for flucytosine, fluconazole, voriconazole, amphotericin-B, and caspofungin.

#### 2.1.3. *Candida* Species-Specific PCR

Fresh subcultures of the isolates were used for DNA extraction by the heat lysis method [7] with slight modifications. Briefly, 2-3 colonies of* Candida* spp. were suspended in 350 *μ*L of molecular grade water and vortexed to make a uniform suspension followed by series of steps including ultrasonication for 2 mins and heating at 80°C for 5 mins followed by centrifugation for 3 mins at 10,000 rpm. The supernatant containing DNA was then subjected to multiplex PCR, targeting the species-specific genes of* Candida* from the internal transcribed spacer (ITS) region. The PCR conditions and reaction composition were modified and optimized at our laboratory from previously described protocol [8]. Briefly, the PCR reaction mix (25 *μ*L) is composed of 12 *μ*L of PCR Ready Mix (Genei, Bangalore, India), 8 *μ*L of molecular grade water, 0.16 *μ*M each of primers, and 5 *μ*L of DNA. The amplicons* C. albicans* (218,110 bp),* C. glabrata* (482 bp),* C. parapsilosis* (229 bp),* C. tropicalis* (218 bp), and* C. krusei* (182 bp) were illuminated under UV light in 3% agarose incorporated with ethidium bromide and analyzed using the gel image analysis software of Bio-Print Mega System (Vilber Lourmat SAS, Marne-la-Vallée, France).

### 2.2. Testing for* In Vitro* Production of Virulence Factors 

#### 2.2.1. Proteinase

Proteinase production was determined using media containing bovine serum albumin as described previously [9]. The presence of halo was observed on staining with amido black. The proteolytic activity (Prz) was calculated as the ratio of diameter of the colony to the total diameter of the colony and the precipitation zone. Isolates with values <1 were considered to have proteolytic activity.

#### 2.2.2. Phospholipase

Phospholipase production of the isolates was determined using egg yolk agar medium as previously described [10]. Once inoculated, the test results were read after 48 hrs. The phospholipase activity (Pz) was measured in a similar manner as the proteinase activity.

#### 2.2.3. Esterase

Esterase production was determined using a medium containing Tween 80 according to earlier reported method [11]. Presence of a halo around the colony when observed against transmitted light was considered as positive for esterase production. The plates were incubated for a maximum of 5 days before terming them nonesterase producers.

#### 2.2.4. Biofilm Production

Microtitre plate assay [12] was used to determine the biofilm producing ability of the isolates. Sabouraud's dextrose broth (6% glucose; pH 6) was used to induce the biofilm production in microtitre plates inoculated with 10^7^ CFU/mL freshly subcultured* Candida* isolates. The plates were incubated at 30°C for 48 hrs, washed with 0.15 M phosphate buffer saline to remove the planktonic cells, and then stained with 0.1% crystal violet, followed by estimation of optical density at 540 nm. For each isolate the biofilm production was tested in triplicate and* C. albicans* ATCC 24433 was used as a standard strain. The strength of biofilm production (weak, moderate, and strong) was calculated using a method previously reported [13].

### 2.3. Statistical Analysis

Statistical software SPSS (ver. 16, IL, Chicago) was used for data analysis. To estimate the frequencies, descriptive statistical tools were used. Measure of agreement (*κ*) between the diagnostic tests was estimated using cross tabulations and kappa (*κ*) value > 0.6 was considered as a test with good agreement with the gold standard, and *χ* ^2^ test was used for determining the association of virulence factors with site of isolation. The significance level was set at a *P* value <0.05.

## 3. Results 

**Figure 1:**
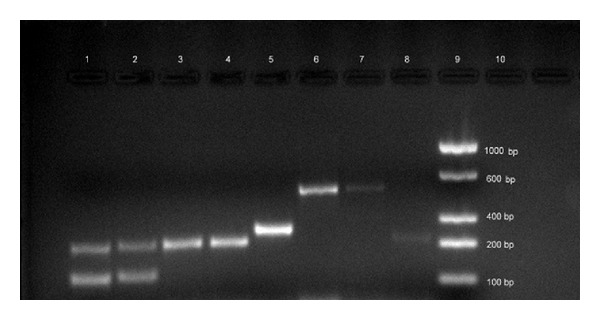
PCR gel electrophoresis of* Candida* spp. Lane 1:* C. albicans* (ATCC 24433), Lane 2:* C. albicans* (clinical isolate), Lane 3:* C. tropicalis* (ATCC 750), Lanes 4 and 8:* C. tropicalis* (clinical isolates), Lane 5:* C. parapsilosis* (clinical isolate), Lane 6:* C. glabrata* (ATCC 2001), Lane 7:* C. glabrata* (clinical isolate), and Lane 9: molecular grade ladder.

Of the total 110 isolates studied, 54 (49%) were obtained from the blood cultures of patients with candidaemia comprising* C. tropicalis* (25, 46%),* C. parapsilosis* (16, 30%),* C. albicans* (7, 13%),* C. glabrata* (2, 4%),* C. krusei* (1, 2%), and other* Candida* spp. (3, 6%). Another 56 (51%)* Candida* isolates were from women of reproductive age group diagnosed with VVC involving* C. albicans* (34, 61%),* C. glabrata* (11, 20%),* C. tropicalis* (5, 9%),* C. parapsilosis* (2, 4%),* C. krusei* (2, 4%), and other* Candida* spp. (2, 4%). All the isolates were primarily identified using species-specific PCR (Figure 1). Among the* Candida* spp. isolates,* C. albicans* (61%) and* C. glabrata* (20%) were most common in VVC, whereas* C. tropicalis* (46%) and* C. parapsilosis* (30%) were most common in BSIs. As PCR did not identify the species of 5* Candida* isolates, only 105* Candida* were included in the analysis of diagnostic utility of HiCrome* Candida* Differential Agar and Vitek 2 Compact system. The sensitivity and specificity of HiCrome* Candida* Differential Agar and Vitek 2 Compact for the species identification of* Candida* are shown in Table 1 and the comparison between the three methods is depicted in Table 2.

The antifungal susceptibility testing was done using the Vitek 2 Compact system for all the 105* Candida* isolates. Isolates were classified as sensitive, intermediately sensitive, and resistant to each antifungal agent based on their minimum inhibitory concentrations (MICs). All the isolates were uniformly susceptible to amphotericin-B and flucytosine in our study.* C. parapsilosis* demonstrated the least susceptibility (64%) to voriconazole followed by* C. tropicalis* (79%). Antifungal susceptibility patterns of study isolates are depicted in Table 3. Isolates from BSIs showed intermediate sensitivity or resistance to various antifungal agents, while fluconazole intermediate susceptibility was seen in two* C. albicans* (2/34, 6%) isolated from VVC.

Of the 105 isolates, 11 had lost viability and hence were not available for further virulence studies. We could study virulence characters of 94 isolates comprising 48* Candida* from blood and 46 from the high-vaginal specimens. Varied production of virulence factors among the two infection groups and various species of* Candida* were observed. Proteinase production was more common among invasive isolates (17, 35%, *P* = 0.017) in comparison with high-vaginal isolates (6, 13%) when tested using *χ* ^2^ test. Phospholipase production was more common among high-vaginal isolates (13, 28%, *P* = 0.001) than in blood isolates (2, 4%). Esterase production and biofilm production were observed in isolates from both infections with no statistically significant difference (*P* > 0.05) (Table 4).

Among the various species tested,* C. albicans* (24/39, 62%) and* C. tropicalis* (18/30, 60%) were the leading producers of biofilm. Comparison of production of virulence factors among* C. albicans* and non-*albicans Candida* did not show a statistically significant difference with respect to the production of biofilm (*P* = 0.295), esterase (*P* = 0.478), and proteinase (*P* = 0.09). Production of phospholipase was significantly higher in* C. albicans* (93%, *P* < 0.001) than in non-*albicans Candida* (7%). Distribution of virulence factors among study isolates based on site of isolation and various species is depicted in Table 4.

Coproduction of proteinase and esterase was observed in 12/48 (25%) of the blood isolates, in comparison with 2/46 (4%) of the high-vaginal isolates (*P* < 0.001). However, production of more than one virulence factor among* C. albicans* and non-*albicans Candida* did not show any statistical significance (14/39 (36%) and 15/55 (27%), *P* = 0.55) on *χ* ^2^ analysis.

## 4. Discussion

Increasing number of treatment failures, associated mortality, and shift to more resistant isolates advocate the need for species identification in* Candida*. Though phenotypic methods for speciation are effective, they lack the rapidity and reproducibility. Utility of molecular techniques like PCR, RFLP, and AFLP is very promising but is expensive restricting their utility to research and reference laboratories [14]. In our study, we used PCR for species identification as the standard method and compared the utilities of Vitek 2 Compact and HiCrome* Candida* Differential Agar in identification of* Candida*. We observed that the sensitivity and specificities of both methods were comparable with each other and PCR. The measure of agreement (*κ*) for Vitek 2 Compact and HiCrome* Candida* Differential Agar with PCR was 0.895 and 0.826, respectively, suggesting the applicability of both methods in routine diagnostic laboratories. The performance of HiCrome* Candida* Differential Agar in our study was comparable to previous reports [15]. However, we observed low sensitivity (79%) of HiCrome Agar in identification of* C. parapsilosis*, which could probably be due to the same color of colony (cream to white) for* C. parapsilosis, C. glabrata*, and* C. kefyr* as instructed by the manufacturer. However, in our study three isolates of* C. parapsilosis* had white colonies with light green centers mimicking the colonies of* C. albicans*. Vitek 2 Compact system was rapid in reporting the identity and antifungal susceptibility results with an average turnaround time of 17 hrs, while HiCrome* Candida* Differential Agar scores better for its general utility as a primary isolation and identification medium from clinical specimen. We also noticed a high interobserver measure of agreement (*κ* = 0.892) for interpreting the results of HiCrome* Candida* Differential Agar indicating high reproducibility.

The present study demonstrates higher isolation rates of non-*albicans Candida* in BSIs possibly due to the involvement of cases from high risk patient care areas.* C. tropicalis* was the most common pathogen isolated from patients with blood stream infections. This finding is in agreement with studies previously reported [13]. We report a higher proportion of* C. albicans* in VVC, which is in contrast to the observations of few studies indicating the changing trends in etiologies with a shift towards non-*albicans Candida*. Geographic differences and increased use of self-medications with antifungal agents might have contributed to upsurge of infections by more resistant* Candida* spp. [16, 17]. However various studies worldwide have shown a predominance of* C. albicans* in mucosal infections and colonization [18, 19].

Sensitivity rates of* C. albicans* for fluconazole, flucytosine, voriconazole, amphotericin-B, and caspofungin were 95%, 100%, 97.5%, 100%, and 97.5%, respectively, and these rates are similar to those reported by Pahwa et al. [20] and higher than the sensitivity rates reported by Chander et al. [4]. The latter group reported a far lower sensitivity rate of* C. albicans* to fluconazole (38%) as the study isolates were obtained from nosocomially acquired candidemia and hence higher resistance to common antifungal agents. However, as was observed in our study, no resistance to amphotericin-B was observed. There are reports of varying rates of resistance to azoles among isolates, primarily non-*albicans Candida* from VVC [21]. However, we did not find antifungal resistance among* Candida* spp. isolated from VVC except intermediate susceptibility of two isolates of* C. albicans* to fluconazole.

Numerous virulence attributes of* Candida* have been postulated that can facilitate the transition of a mucosal colonizer to a fatal disseminating pathogen. While adhesions help the yeast in adhering to the host cell surfaces, hydrolytic enzymes like proteinases, phospholipases, and lipases along with hyphal forms promote the penetration through cells. In addition to these virulence attributes of* Candida*, host immunity plays a major role in restricting these infections to either a localized form as seen in immunocompetent hosts or a deep-seated and disseminating form of infections common among immunocompromised hosts [22].

An emphasis on hydrolytic enzymes produced by* Candida* spp. can help in understanding the disease process better as these enzymes have activity on a wide array of host substrates. Secreted aspartyl proteinases (SAP) in* Candida* are known to enhance the hypha formation, epithelial cell damage, invasion, and inflammatory responses.* In vivo* experimental models also demonstrated an increase in the invasiveness of yeasts with the production of proteinases [22, 23]. An association between production of SAP and increased hyphae formation* in vivo* has been reported previously [22]. Considering the coregulation of SAP and hyphal production in* Candida* spp., we assume the isolates from BSIs to be predominantly proteinase producers. In our study, we observed a statistically significant increase in the production of proteinases among the blood isolates compared to those from VVC. This finding is in concordance with the previous findings of Costa et al. [24]. The production of proteinases was significantly high among* C. tropicalis*, possibly contributing to its increased occurrence in BSIs. Though* C. albicans* is the leading producer of proteinases among all the species of* Candida*, recent studies suggest the production of 4 types of SAP by* C. tropicalis* [25].

Phospholipases are a group of enzymes produced by* Candida* species that primarily help in digesting the phospholipids of the host cells leading to cell lysis.* C. albicans* is the major producer of phospholipases, whereas a less proportion of non-*albicans Candida* produce this enzyme. It is postulated that more sensitive methods are needed to detect the lesser amount of phospholipases produced by non-*albicans Candida* [26]. In our study, the majority (93%) of phospholipase producers were* C. albicans*, while only 7% of non-*albicans Candida* were positive for phospholipases. Phospholipase production was predominately seen in vaginal isolates possibly due to higher number of* C. albicans* in this group.

Though production of esterase has been documented as virulence character among clinical isolates of* Candida*, little is known about the pathogenesis. Recent investigations suggest the mechanism of virulence is due to the cytotoxic effects of lipases and esterase in the host tissues [27]. However, we did not find a significant difference in esterase production among the invasive and noninvasive isolates. The proportion of esterase positive isolates in this study was lower in comparison to few published reports [28, 29].

Biofilm production is considered as one of the most potent pathogenic traits attributed to treatment failures and recurrent infections. We report similar rates of biofilm production among blood and mucosal isolates (52% and 54%), but higher biofilm production was seen in* C. albicans* as compared to non-*albicans Candida* [13]. Though the exact reason for the higher biofilm production rates by* C. albicans* is not completely understood, studies using the scanning electron microscopy to understand the complex biofilm architectures have attributed the integrity and strength of these biofilms to the higher number of hyphal elements produced by* C. albicans* than* C. tropicalis* and* C. parapsilosis*. The latter two species form biofilms of lesser strength and the biofilm in these two species is primarily constituted of micro colony aggregates of yeast cells [27]. Considering the need of high glucose concentrations for non-*albicans Candida* to produce biofilms* in vitro* we used SDB with 6% glucose [30]. We anticipated high glucose concentration to simulate* in vivo* conditions for non-*albicans Candida* thus promoting the production of detectable biofilms* in vitro*. Notably, biofilm production of* C. tropicalis* (62%) was comparable to* C. albicans* (60%) in our study. With an increasing trend of blood stream infections due to* C. tropicalis*, further studies to understand its biofilm architecture and antifungal susceptibility are warranted.

Our study has few limitations. We could not compare the production of virulence characters among the same species isolated from different sites due to small number of few* Candida* species. However, current information might be useful for a better understanding of the role of host environmental factors and individual virulence factors in site specific disease development. Similarly, interspecies comparison of virulence mechanisms among all the pathogenic species of* Candida* could not be achieved in the current study due to the small numbers of few species of* Candida*.

However, our study involved common* Candida* isolates form invasive and mucosal infection sites and analyzed the production of important virulence determinants, especially among less explored non-*albicans Candida*. More sensitive methods are needed to detect certain enzymes like esterases in these isolates. Exploring genetic mechanisms of virulence in non-*albicans Candida* would help in understanding the spectrum of disease and changing trends in disease epidemiology.

## 5. Conclusions

From this study, we found* C. albicans* followed by* C. glabrata* were the leading pathogens causing VVC, while* C. tropicalis* and* C. parapsilosis* were predominant in blood stream infections. Vitek 2 Compact and HiCrome Differential Agar have proven their efficacy in speciation of* Candida*; however incorporation of either or both techniques in daily practice depends on the throughput and resource settings of the laboratory. We also observed an overall increase in the production of virulence characters among non-*albicans Candida*, especially* C. tropicalis*. Most of* C. albicans* in our study were susceptible to fluconazole, favoring its use in empiric therapy of vaginal infection. Resistance to various commonly used antifungal agents among non-*albicans Candida* suggests the need for species identification in routine laboratories for initiation of appropriate antifungal therapy.

## Figures and Tables

**Table 1:** Diagnostic accuracies of Vitek 2 Compact and HiCrome Agar in comparison with PCR.

Species (*N*)∗ (Total = 105)	Vitek 2 Compact		HiCrome Agar	
	Sensitivity (%)	Specificity (%)	Sensitivity (%)	Specificity (%)
	Confidence intervals (95%)		Confidence intervals (95%)	
*C. albicans* (41)	93 (81–98)	100 (94–100)	100 (91–100)	91 (83–96)
*C. tropicalis* (30)	100 (88–100)	95 (88–98)	88 (72–96)	100 (95–100)
*C. parapsilosis* (18)	100 (82–100)	98.8 (94–99)	79 (72–100)	100 (96–100)
*C. glabrata* (13)	100 (75–100)	100 (96–100)	100 (76–100)	100 (92–99)
*C. krusei* (3)	100 (30–100)	100 (96–100)	100 (30–100)	100 (96–100)

Measure of agreement (*κ*)	0.895		0.826	

*Number of isolates identified using the species-specific PCR (reference method for speciation in our study).

**Table 2:** Identification of *Candida* spp. by using species-specific PCR, Vitek 2 Compact, and HiCrome Agar.

*Candida* spp.	Species-specific PCR (*N*)	Vitek 2 Compact (*n*) (misidentified as)	HiCrome Agar (*n*) (misidentified as)
*C. albicans *	41	37 (3 *C. tropicalis*, 1 *C. parapsilosis*)	48 (4 *C. tropicalis*, 3 *C. parapsilosis*)
*C. tropicalis *	30	27 ( 2 *C. parapsilosis*, 1 *C. albicans*)	25 (1 *C. parapsilosis*, 1 *C. glabrata*)
*C. parapsilosis *	18	17 (1 *C. tropicalis*)	13 (1 *C. tropicalis*)
*C. glabrata *	13	13	12
*C. krusei *	3	3	3
Unidentified∗	5	5	0

*Of the five isolates unidentified by PCR, two were identified as *Trichosporon inkin* by Vitek 2 Compact. The remaining three were unidentified by both PCR and Vitek 2 Compact.

**Table 3:** Antifungal susceptibility patterns of *Candida* spp.

Antifungal agent	Fluconazole			Flucytosine∗			Voriconazole			Amphotericin-B∗			Caspofungin		
Susceptibility pattern *n* (%)	S	I	R	S	I	R	S	I	R	S	I	R	S	I	R

CLSI∗∗ breakpoints (*μ*g/mL)	≤2	4	≥8	≤4	8–16	≥32	≤0.12	0.25–0.5	≥1	≤4	8–16	≥32	≤0.25	0.5	≥1

*C. albicans* (*N* = 41)	39 (95)	2 (5)	—	41 (100)	—	—	40 (97.5)	1 (2.5)	—	41 (100)	—	—	40 (97.5)	—	1 (2.5)
*C. tropicalis* (*N* = 29)	29 (100)	—	—	29 (100)	—	—	23 (79)	6 (21)	—	29 (100)	—	—	28 (96.5)	—	1 (3.5)
*C. parapsilosis* (*N* = 19)	19 (100)	—	—	19 (100)	—	—	12 (64)	6 (31)	1 (5)	19 (100)	—	—	19 (100)	—	—
*C. glabrata* ∗ (*N* = 13)	13 (100)	—	—	13 (100)	—	—	12 (92)	1 (8)	—	13 (100)	—	—	13 (100)	—	—
*C. krusei* (*N* = 3)	—	—	R∗	3 (100)	—	—	3 (100)	—	—	3 (100)	—	—	3 (100)	—	—

S: sensitive; I: intermediate; R: resistant.

Susceptibility patterns presented as frequency (%).

∗∗MIC breakpoints of YST006 AFT cards used in Vitek 2 Compact system. Interpretation based on CLSI M27-S4 Volume 32.

∗MIC breakpoints for *Candida* spp.

R∗ Intrinsic resistance.

**Table 4:** Production of various virulence factors among *Candida* spp.

Virulence factors	*C. albicans* (*N* = 39) *n* (%)	*C. tropicalis* (*N* = 30) *n* (%)	*C. parapsilosis* (13) *n* (%)	*C. glabrata* (9) *n* (%)	*C. krusei* (3) *n* (%)	Cumulative non-*albicans Candida* (55)	*P* value	Blood *n* (%) total = 48	High-vaginal *n* (%) total = 46	*P* value
Proteinase	6 (15)	14 (48)	0	2 (22)	1 (33)	17 (31)	0.095	17 (35)	6 (13)	**0.017**
Phospholipase	14 (36)	0	1 (7)	0	0	1 (2)	**<0.01**	2 (4)	13 (28)	**0.001**
Esterase	8 (20)	13 (45)	2 (14)	0	0	15 (27)	0.478	15 (31)	8 (17)	0.103
Biofilm production	24 (60)	18 (62)	5 (35)	2 (22)	0	25 (45)	0.295	24 (52)	25 (54)	0.530
